# Non-coding RNAs in cardiac regeneration

**DOI:** 10.18632/oncotarget.6073

**Published:** 2015-10-10

**Authors:** Lichan Tao, Yihua Bei, Yanli Zhou, Junjie Xiao, Xinli Li

**Affiliations:** ^1^ Department of Cardiology, The First Affiliated Hospital of Nanjing Medical University, Nanjing, China; ^2^ Regeneration and Ageing Lab, Experimental Center of Life Sciences, School of Life Science, Shanghai University, Shanghai, China; ^3^ Shanghai Key Laboratory of Bio-Energy Crops, School of Life Science, Shanghai University, Shanghai, China

**Keywords:** non-coding RNA, cardiac regeneration, microRNA, long non-coding RNA

## Abstract

Developing new therapeutic strategies which could enhance cardiomyocyte regenerative capacity is of significant clinical importance. Though promising, methods to promote cardiac regeneration have had limited success due to the weak regenerative capacity of the adult mammalian heart. Non-coding RNAs (ncRNAs), including microRNAs (miRNAs, miRs) and long non-coding RNAs (lncRNAs), are functional RNA molecules without a protein coding function that have been reported to engage in cardiac regeneration and repair. In light of current regenerative strategies, the regulatory effects of ncRNAs can be categorized as follows: cardiac proliferation, cardiac differentiation, cardiac survival and cardiac reprogramming. miR-590, miR-199a, miR-17-92 cluster, miR302-367 cluster and miR-222 have been reported to promote cardiomyocyte proliferation while miR-1 and miR-133 suppress that. miR-499 and miR-1 promote the differentiation of cardiac progenitors into cardiomyocyte while miR-133 and H19 inhibit that. miR-21, miR-24, miR-221, miR-199a and miR-155 improve cardiac survival while miR-34a, miR-1 and miR-320 exhibit opposite effects. miR-1, miR-133, miR-208 and miR-499 are capable of reprogramming fibroblasts to cardiomyocyte-like cells and miR-284, miR-302, miR-93, miR-106b and lncRNA-ST8SIA3 are able to enhace cardiac reprogramming. Exploring non-coding RNA-based methods to enhance cardiac regeneration would be instrumental for devising new effective therapies against cardiovascular diseases.

## INTRODUCTION

Cardiovascular disease (CVD) represents a major cause of morbidity and mortality worldwide [1]. Despite continuous advances in current clinical therapies, CVD is still considered to be a major global epidemic in need of innovative therapeutics [2]. In light of this, extraordinary efforts have been devoted to the study of novel cellular mechanisms characterizing cardiac physiology and pathology. Recently, cardiac repair has attracted significant interest as understanding its underlying mechanism may provide new avenues for fatal CVD.

Unlike amphibians and fish, the majority of cardiomyocytes in the mammalian heart lose the capacity to proliferate after birth [3, 4]. Recently, mounting evidence suggests that developing hearts have a strong growth and regenerative capacity arising from either differentiation of cardiac progenitor cells (CPCs) or proliferation of cardiomyocytes [5–7]. Of note, nearly half of cardiomyocytes are replaced during a whole human lifespan [3]. In the normal mouse heart, cardiomyocyte turnover occurs mainly through activation of resident cardiomyocytes at a rate of ~1.3-4%/year [8]. After myocardial injury, the rate of cardiomyocyte renewal may increase significantly, especially in the infarct border zone [9]. However, during a significant myocardial injury, a large number of cardiomyocytes are lost and may lead to heart failure, as the cardiac regenerative response is often insufficient to replace the lost cardiomyocytes. This ultimately leads to cardiac dysfunction, poor contractile function and massive fibrosis [10]. Cardiac repair is extremely limited due to the weak capacity of the adult mammalian heart to regenerate. Therefore, developing new therapeutic strategies which could enhance cardiomyocyte regenerative capacity is of significant clinical importance.

Non-coding RNAs (ncRNAs) are functional RNA molecules without protein coding functions [11]. ncRNAs can be divided into small (<200 nt) non-coding RNAs and longer RNAs (>200 nt). Small ncRNAs include microRNAs (miRs), transfer RNAs, and small nucleolar RNAs. Longer ncRNAs include ribosomal RNAs, natural antisense transcripts and other long non-coding RNAs (lncRNAs) [12]. Recently, the function of many of the newly identified ncRNAs has been validated, including the identification of miRs and lncRNAs involved in cardiac regeneration and repair [13].

## CARDIAC REGENERATION

Certain fish and amphibians retain a strong capacity for cardiac regeneration throughout their whole life [14–16]. For example, the zebrafish can fully regenerate its heart after surgical amputation of the cardiac apex and can tolerate injury of up to 20% of the ventricular mass [17, 18]. Using a *Cre/loxp* system to trace the lineage of cardiomyocytes in the adult fish, studies demonstrate that newly-formed cardiomyocytes are derived from the division of differentiated cardiomyocytes through increased expression of polo-like kinase 1 (plk1) [19]. Although mammalian hearts lack the robust regenerative capacity as observed in the zebrafish, postnatal mammalian hearts also experience a degree of cardiomyocyte renewal in physiological or pathological conditions [20,21]. To detect the origin of mammalian cardiomyocyte renewal, a study combining two lineage tracing approaches, genetic fate-mapping with isotope labeling and multi-isotope imaging mass spectrometry, reported murine cardiomyocyte genesis occurs at a very low rate and mainly derives from the differentiation of pre-existing cardiomyocytes in both the normal ageing process and in myocardial injury. Interestingly, the rate of cardiomyocyte renewal is significantly increased adjacent to areas of myocardial injury [22].

In addition to division of pre-existing cardiomyocytes, progenitor/stem cells also contribute to cardiomyocyte renewal [8, 23–25]. A study using genetic fate mapping in conditional green fluorescent protein (GFP)-labeled transgenic mice (cardiomyocytes are GFP+ and stem or precursor cells are GFP-) revealed that during normal ageing, the percentage of GFP+ cardiomyocytes remained unchanged. This finding indicates cardiomyocyte turnover occurs mainly through differentiation of resident cardiomyocytes, found to be at a rate of ~1.3-4%/year [8]. However, in injured hearts, especially myocardial infarction, the number of GFP- cardiomyocytes increased and the percentage of GFP+ cardiomyocytes decreased. This suggests that stem or precursor cells replace injured cardiomyocytes at a significant rate [26].

Despite these observations, cardiac regeneration capacity is still limited due to the extremely low rate of cardiomyocyte production in the adult heart. Thus, it is of great clinical importance to understand the cellular and molecular mechanisms underlying cardiac regeneration. Overall, there are three strategies to induce cardiac regeneration in the adult heart: (1) transplant exogenous progenitor/stem cells to damaged myocardium, (2) promote resident progenitor/stem cells to differentiate into mature cardiomyocytes, and (3) enhance the proliferation of pre-existing cardiomyocytes. For strategies 1 and 2, multiple studies have used adult stem cells, pluripotent stem cells (iPSCs), or cellular reprogramming to protect the injured heart [7, 20, 27, 28]. For example, in a GFP transgenic mouse model of myocardial injury, cell therapy with bone marrow-derived c-kit+ cells diluted the GFP+ cardiomyocyte pool and ultimately improved cardiac function, suggesting that there is transdifferentiation or cell fusion of exogenous c-kit+ cells to cardiomyocytes with resulting improved functionality [29]. Other studies indicate that heart failure (HF)-derived bone marrow multipotent mesenchymal stromal cells (BM-MMSCs) demonstrate an early decrease of proliferative capacity, they also upregulate genes that control regeneration in addition to fibrosis. However, low density seeding in combination with moderate hypoxia results in improved regeneration and expansion of BM-MMSCs as well as prevention of lost replication potential, thus (HF)-derived BM-MMSCs can also be applied to cell therapy by changing culture condition [30]. For strategy 3, enhancing the endogenous signaling pathway of cardiomyocyte regeneration is also of significant importance. For example, growth factor neuregulin1 (NRG1) and its tyrosine kinase receptor (ErbB4) are reported to regulate cardiac regeneration by inducing cardiomyocyte proliferation [31]. Genetically activating ErbB4 and pharmacologically injecting NRG1 in adult mice induces cardiomyocyte cell-cycle activity to promotes cardiac regeneration and protects against myocardial injury [32]. Physiological exercise is another way to control cardiac regeneration, cardiomyocyte size, and apoptosis by regulating IGF-1-PI3k-Akt or other signaling pathways [33, 34]. Despite extensive studies, the progress in cardiac regeneration has been slow.

The regenerative rate in the human heart is significantly lower than in mice or rats. Recently, a study reports that the number of cardiomyocytes remains constant during the whole human life span. Cardiomyocyte turnover decreases with age and is <1% in the human adult heart [35]. Thus, there is an urgent need for new therapeutic strategies to enhance cardiomyocyte regenerative capacity.

## NON-CODING RNA IN CARDIAC REGENERATION (FIGURE 1)

Currently, emerging evidence indicates that non-coding RNAs (ncRNAs) are responsible for specialized biological processes during cardiac development, disease and ultimately repair, such as transcriptional regulation, post-transcriptional gene control, epigenetic control, and nuclear genome organization [35–37]. In this context, incorporating ncRNAs within cardiac gene regulatory networks may provide a new opportunity for therapeutic intervention via regenerative medicine in the heart, especially miRs or lncRNAs. In light of current regenerative strategies, the regulatory effects of ncRNAs can be categorized as follows: cardiac proliferation, cardiac differentiation, cardiac survival and cardiac reprogramming.

**Figure 1:**
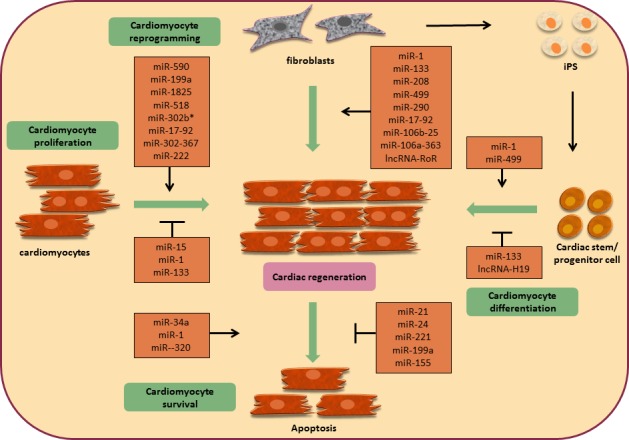
Non-coding RNAs in cardiac regeneration

## CARDIOMYOCYTE PROLIFERATION

In a high-throughput functional screen for human miRNAs that enhance neonatal cardiomyocyte proliferation, 204 miRNAs were identified that increased proliferation over 2-fold compared to control [38]. Among these, miR-590 and miR-199a were studied in further detail and shown to activate the cell cycle and promote cardiomyocyte proliferation both *in vitro* and *in vivo*. Interestingly, during myocardial infarction, these two miRNAs not only stimulate cardiac regeneration, but also preserve cardiac function and decrease cardiac fibrosis [38]. Other miRNAs, such as miR-1825, miR-518, miR-302b*, have all been screened and validated by EdU, ki67, and phospho-histone H3 staining in rat or mouse cardiomyocytes, but the specific effects of these miRNAs on the heart require further study.

The miR-17-92 cluster has also been identified as a critical regulator of cardiac proliferation [39]. Transgenic overexpression of the miR-17-92 cluster in the heart induces cardiomyocyte proliferation in embryonic, postnatal, and even adult hearts by inhibiting phosphatase and tensin homolog (PTEN). Importantly, overexpression of the miR-17-92 cluster reverses cardiac dysfunction and cardiac fibrosis after myocardial infarction [39]. Another microRNA cluster, miR302-367, also promotes cardiomyocyte proliferation during developmental and adult stages [40]. Transient overexpression of miR302-367 leads to increased cardiomyocyte proliferation, decreased cardiac fibrosis and improved cardiac function after injury by targeting the Hippo signaling pathway.

As demonstrated previously, exercise induces physiological cardiac growth and protects against myocardial injury [41–43]. miR-222, upregulated in two distinct models of exercise (running and swimming), is necessary for exercise-induced cardiac proliferation due to its role in inhibiting cyclin-dependent kinase inhibitor 1B (p27), homeodomain-interacting protein kinase 1 (HIPK1) and homeobox containing 1 (HMBOX1) [44]. Overexpression of miR-222 in the heart confers resistance to cardiac remodeling and dysfunction after ischemia/reperfusion injury, while inhibition of miR-222 blocks cardiac growth in response to exercise [44]. This study links a specific miRNA with exercise as well as ischemia and holds great promise for targeting cardiomyocyte proliferation.

In addition to miRNAs that enhance cardiomyocyte proliferation, there are also miRNAs that suppress cardiac regeneration. The miR-15 family, regulated in the mouse heart at 1 and 10 days of age, suppresses numerous cell cycle genes and mediates postnatal cell cycle arrest [45]. Importantly, the miR-15 family modulates neonatal heart regeneration by repressing postnatal cardiomyocyte proliferation, thus leading to postnatal loss of cardiac regenerative capacity [46]. Inhibition of the miR-15 family from the early postnatal to adult stages increases cardiomyocyte proliferation and improves left ventricular function after myocardial infarction [46, 47].

miR-1 and miR-133 are both expressed in cardiac and skeletal muscle and regulated by the transcription factors MyoD, myocyte enhancer factor-2 (Mef2) and serum response factor (SRF) [48]. *In vivo* overexpression of miR-1 in the mouse during development inhibits cardiomyocyte proliferation by suppressing the transcription factor heart and neural crest derivatives-expressed protein 2 (Hand2) [48–50]. Deletion of miR-133a (involving both miR-133a-1 and miR-133a-2) results in up-regulation of smooth muscle genes in the heart and aberrant cardiomyocyte proliferation as detected by enhanced phospho-histone H3 staining [51, 52]. Overexpression of miR-133a results in diminished cardiomyocyte proliferation, which is consistent with the conclusion that miR-133a represses cardiac proliferation [53].

It is well known that lncRNA can control proliferation and apoptosis in various cell types; however, knowledge regarding its role in controlling cardiomyocyte proliferation remains limited. Therefore, researching the effects of lncRNA on cardiomyocyte proliferation may attract significant interest in the future. Importantly, studies show that enhanced cardiomyocyte proliferation is associated with protection against myocardial injury, suggesting that non-coding RNAs may be therapeutic targets for heart regeneration and cardiac repair.

## CARDIOMYOCYTE DIFFERENTIATION

miRNAs are reported to play important roles in the emergence of cardiac progenitors that trans-differentiate to cardiomyocytes as well as maintenance of the differentiated state in cardiomyocytes [54, 55]. miR-1 is a critical regulator of muscle cell proliferation and differentiation [56]. Overexpression of miR-1 in the developing heart can lead to growth retardation at embryonic day 13.5, thinner ventricular walls and ultimately heart failure results due to decreased cardiomyocyte proliferation by suppressing Hand2 [49]. Deletion of miR-1 (miR-1-2) leads to numerous defects in cardiogenesis, cardiac conduction and the cell cycle [48]. Thus, gain-of-function and loss-of-function studies demonstrate a key role of miR-1 in inhibiting cell proliferation and promoting differentiation. *In vitro* overexpression of miR-1 upregulates the cardiac differentiation genes nk2 homeobox 5 (NKX2.5) and β-MHC during mouse and human embryonic stem cell (ESC) differentiation [57, 58]. Importantly, transplantation of mouse ESCs overexpressing miR-1 to the border zone of myocardial infarction protects against ischemia-induced apoptosis by activating the PTEN-AKT signaling pathway [59]. These studies confirm miR-1 promotes cardiomyocyte differentiation and reprogramming. miR-1 and miR-133 are transcribed together as they are polycistronically clustered on the same chromosome. However, unlike miR-1 [51], miR-133 appears to inhibit cardiac differentiation. Overexpression of miR-133 suppresses cardiac markers in mouse and human ESCs, and in the absence of miR-133, myocytes proliferate partly through activation of SRF and G1/S-specific cyclin-D2 (CyclinD2) [51, 56], highlighting the inhibitory role of miR-133 in both cardiac proliferation and differentiation.

miR-499, enriched in adult progenitor cells in the heart, is also strongly associated with cardiac differentiation. Similar to miR-1, overexpression of miR-499 reduces cardiac progenitor cell proliferation and promotes the formation of beating embryoid bodies of mouse ESCs by up-regulating GATA4 and Mlc-2v [58]. In human cardiomyocyte progenitor cells (hCPCs), cardiac differentiation is greatly enhanced by increasing spontaneous beating areas of differentiated cells after transfecting with miR-499. In addition, cardiomyocyte-specific genes such as cardiac troponin T, and Mef2 are also up-regulated [50]. *In vivo* transplantation of human cardiac stem cells (hCSCs) transfected with miR-499 to the border zone of ischemic hearts promotes transformation of hCSCs to mature cardiomyocytes [60]. Overall, these *in vitro* and *in vivo* studies demonstrate the role of miR-499 in promoting cardiac differentiation.

A long non-coding RNA, H19, has been reported to negatively regulate body weight and cell proliferation [61]. Recently, down-regulation of H19 has been shown to promote differentiation of parthenogenetic embryonic stem cells (p-ESCs) to cardiomyocytes with strong heart-like beating, supporting the notion that reduction of H19 improves p-ESCs lineage determination to mesoderm cells such as cardiomyocytes [62, 63].

Together, these studies show that ncRNAs help balance cardiomyocyte differentiation with proliferation. These findings may be translated clinically by using ncRNAs to induce transferred stem cells or resident cardiac progenitor cells to differentiate into mature cardiomyocytes and replace damaged myocardium [16].

## CARDIOMYOCYTE SURVIVAL

Although there have been great advances in stem cell therapy for cardiac injury, the low survival rate of transplanted cells in injured myocardium remains a major problem [64]. Therefore, the development of novel treatment strategies to boost cell survival is of significant importance.

miR-21 is decreased within the infarcted area in myocardial infarction (MI), indicating a protective effect of miR-21 on cardiac survival [65]. *In vitro* gain-of-function and loss-of-function studies of miR-21 confirm that it protects against ischemia-induced cardiomyocytes apoptosis by inhibiting programmed cell death protein 4 (PDCD4) and activator protein 1 (AP-1). Overexpression of miR-21 by adenovirus (Ad-miR-21) in rats decreases cardiac fibrosis by 29% at 24 hours, reduces ventricular dimension at 2 weeks and decreases cell apoptosis in the infarct or border area after acute myocardial infarction (MI) [65, 66]. These studies indicate that miR-21 promotes cardiac survival and apoptosis. miR-24 shares similar functions with miR-21, reducing cardiac apoptosis and increasing cell number by 53% through the repression of BH3-domain-containing protein (Bim). In a mouse MI model, overexpression of miR-24 is protective against cardiac apoptosis as partially mediated by Bim [67]. Interestingly, a study demonstrated that CPCs transfected with a cocktail of 3 microRNAs (miR-21, miR-24 and miR-221) exhibit higher viability compared to untreated CPCs when challenged with serum free medium. In a MI model, intramyocardial injection of CPCs transfected with miRNAs decreases cardiac dysfunction by regulating Bim [68]. In summary, the cocktail of microRNAs (miR-21, miR-24, and miR-221) can greatly boost the survival of transplanted cardiac progenitor cells, thus improving cardiac function.

In a hypoxia preconditioning environment, miR-199a is acutely downregulated in cardiomyocytes through up-regulation of hypoxia-inducible factor (Hif)-1 [69, 70]. Overexpression of miR-199a during hypoxia reduces apoptosis mediated by Hif-1 inhibition and its stabilization of p53, whereas inhibition of miR-199a recapitulates the hypoxic environment by up-regulating Hif-1 and Sirtuin 1(Sirt1). These data indicate that miR-199a plays a critical role in regulating the hypoxia-related pathway and decreases apoptosis in the setting of hypoxic damage. Another miRNA, miR-155, also prevents necrotic cell death in human cardiomyocyte progenitors by 40±2.3% by repressing receptor interacting protein 1 (RIP1) [71].

In addition to miRNAs that promote cardiomyocyte/progenitor cell survival, there are also several miRNAs that exert the opposite effect on cardiomyocytes. miR-34a is induced in the ageing heart, a state that is a major risk factor for cardiovascular disease and may contribute to worse outcomes in patients with MI [72]. *In vivo* overexpression of miR-34a contributes to cardiac dysfunction in an age-dependent manner. However, silencing or genetic deletion of miR-34a decreases telomere shortening, DNA damage response and cardiomyocyte death induced by ageing. Inhibition of miR-34a can also reduce cardiac fibrosis and improve cardiac function in mice with MI by activating serine/threonine-protein phosphatase 1 regulatory subunit 10 (PNUTS). miR-1, which plays an important role in cardiomyocyte proliferation and differentiation, also plays a key role in regulating cardiac survival [73]. miR-1 transgenic mice have more severe cardiac ischemia/reperfusion (IR) injury as measured by increased LDH, CK levels, cleaved-caspase3 expression and cardiac infarct areas. In contrast, downregulation of miR-1 has an opposite effect. Studies reveal that miR-1 inhibits protein kinase C epsilon (PKC) and heat shock protein 60 (HSP60) in cardiac injury [73]. miR-320 is significantly increased in hearts with I/R injury both *in vivo* and *in vitro* [74]. In turn, gain-of-function of miR-320 results in increased infarction size and apoptosis in hearts with I/R injury both *in vivo* and *ex vivo*. Conversely, administration of antagomir-320 reduces cell apoptosis and infarction size relative to controls. Heat-shock protein 20 (Hsp20), a well-known cardioprotective protein, is a candidate target for miR-320. Downregulation of miR-29 protects the heart against IR injury by decreasing the expression of pro-apoptotic molecular Bax and increasing anti-apoptotic molecular Bcl2 [75].

In summary, these studies indicate that manipulating miRNA levels may be a novel therapeutic strategy for cardiovascular disease mediated by injury-induced apoptosis.

## CARDIOMYOCYTE REPROGRAMMING

Although there have been great advance in cardiac regenerative treatment, repopulation of the injured myocardium with new, functional cardiomyocytes remains a daunting challenge. Recently, landmark reports have raised the possibility of utilizing cellular reprogramming for cardiac regeneration [76–80].

Recently, a study identified a combination of miRNAs (miR-1, miR-133, miR-208 and miR-499) capable of reprogramming fibroblasts to cardiomyocyte-like cells *in vitro*, an effect which is enhanced 10-fold after the administration of janus kinase (JAK) inhibitor [79]. The myocyte-like cells possess morphological and functional properties of mature cardiomyocytes. Importantly, administration of miRNAs to injured myocardium *in vivo* results in the direct reprogramming of cardiac fibroblasts to cardiomyocytes, further confirmed by genetic tracing analysis using *Fsp1Cre*-mice. Enhanced cardiac reprogramming also decreased cardiac fibrosis and improved cardiac function, as indicated by fractional shortening, ejection fraction and other measures of ventricular function [81].

iPS cells were firstly established by overexpressing the four reprogramming factors Oct3/4, Sox2, Klf4, and c-Myc in fibroblasts [82, 83]. The miR-290 family is highly expressed in ESCs and significantly increases the efficiency of reprogramming in mouse embryonic fibroblasts (MEFs). Conversely, antagonizing miR-290 inhibits reprogramming. Within the miR-290 family, miR-284 enhances the efficiency to 75% of that achieved by the three reprogramming factors (Oct3/4, Sox2, and Klf4) alone [84]. Members of the miR-302 family share a similar sequence with miR-290 and also increase the programming efficiency of fibroblasts to mature cardiomyocytes [85–87]. Reportedly, three miR clusters, miR-17-92, miR-106b-25, and the miR-106a-363, are highly expressed when reprogramming fibroblasts to cardiomyocytes. Upregulation of the miR-106b-25 cluster members miR-93 and miR-106b increase cell reprogramming of iPS in combination with three (Oct3/4, Sox2, and Klf4) or four (Oct3/4, Sox2, Klf4, and c-Myc) reprogramming factors [88].

Recently, lncRNA-ST8SIA3 (also named lncRNA-ROR, regulator of reprogramming), is upregulated during iPS generation and enhances the reprogramming of fibroblasts to iPSs or cardiomyocytes [89]. LncRNAs also regulate the pluripotent state in mouse ESCs as evidenced by genome-wide mapping studies [90]. Transcription factors such as Oct4 and Nanog bind promoters of lncRNA. To further elucidate the biological functions of lncRNA expressed in ESCs, loss-of-function studies have been performed. By using a shRNAi high-throughput screen, one study identified 26 lncRNAs required for maintaining the pluripotent state of ESCs; notably, inhibition of these 26 lncRNAs led to loss of pluripotency in ESCs [91]. Therefore, lncRNA provides a new approach in the regulation of reprogramming pluripotent stem cells or fibroblasts to cardiomyocytes.

Overall, recent studies have established that fibroblasts or iPSs can be reprogrammed into mature cardiomyocytes by miRNAs or lncRNA in combination with lineage-significant reprogramming factors, an approach which holds tremendous potential for cardiac regeneration and repair.

## CONCLUSIONS

The adult mammalian heart has a limited capacity to regenerate during ageing or myocardial injury. As a consequence, the endogenous renewal rate is insufficient to recover cardiomyocyte loss and to retain cardiac function under physiological or pathological conditions [92]. Recently, many newly identified ncRNAs have been found to regulate cardiac regeneration. Therefore, it is important to understand the detailed function and molecular mechanism of non-coding RNAs implicated in cardiac renewal and cardiac repair. In this review, we elucidate several ncRNAs participating in different aspects of cardiac regeneration: cardiac proliferation, differentiation, survival and reprogramming. Various studies have provided convincing evidence that miRNAs or lncRNAs may improve cell therapy or endogenous cardiac repair. However, the development of ncRNA therapy still remains challenging.

miRNA/lncRNA-based treatments is a promising emerging field in medicine [93]. One miRNA can control hundreds of genes, and in turn, one gene may be regulated by several miRNAs. Therefore, a major problem is understanding the regulatory networks between miRNAs and mRNAs. In addition, the underlying mechanisms of miRNAs in modulating cardiac regeneration need to be further explored. In addition, miRNAs and lncRNAs are distributed in several tissues and different cell types, and administration of miRNA chemical compound may lead to undesired side effects in other tissues and cells. Thus, cell type or tissue-specific strategies may be needed in future studies. Several miRNAs or lncRNAs are reported to enhance cardiac reprogramming from fibroblast or iPS to mature cardiomyocyte. However, the maturity and heterogeneity of stem cell or fibroblast derived cardiomyocytes and the low survival and retention of delivered cells remain therapeutic concerns.

Although many challenges remain, non-coding RNAs hold tremendous promise for therapeutic cardiac regeneration and repair.
